# Bioaccessibility of Marine Carotenoids

**DOI:** 10.3390/md16100397

**Published:** 2018-10-22

**Authors:** Isabel Viera, Antonio Pérez-Gálvez, María Roca

**Affiliations:** Food Phytochemistry Department, Instituto de la Grasa (CSIC), University Campus, Building 46, Carretera de Utrera km. 1., 41013 Sevilla, Spain; iviera@ig.csic.es (I.V.); aperez@ig.csic.es (A.P.-G.)

**Keywords:** aquaculture, astaxanthin, bioaccessibility, fucoxanthin, humans, marine carotenoids

## Abstract

The benefit of carotenoids to human health is undeniable and consequently, their use for this purpose is growing rapidly. Additionally, the nutraceutical properties of carotenoids have attracted attention of the food industry, especially in a new market area, the ‘cosmeceuticals.’ Marine organisms (microalgae, seaweeds, animals, etc.) are a rich source of carotenoids, with optimal properties for industrial production and biotechnological manipulation. Consequently, several papers have reviewed the analysis, characterization, extraction and determination methods, biological functions and industrial applications. But, now, the bioaccessibility and bioactivity of marine carotenoids has not been focused of any review, although important achievements have been published. The specific and diverse characteristic of the marine matrix determines the bioavailability of carotenoids, some of them unique in the nature. Considering the importance of the bioavailability not just from the health and nutritional point of view but also to the food and pharmaceutical industry, we consider that the present review responds to an actual demand.

## 1. Introduction

Carotenoids are a family of natural pigments extensively present in the vegetal kingdom, photosynthetic microorganisms and some fungi, which mains function are their contribution to the light-harvesting process, the filtering of deleterious light radiations and the antioxidant activity. Structurally, carotenoids are isoprenoid compounds that are classified as *carotenes* (pure hydrocarbons) or *xanthophylls* (oxygenated carotenes). Both families are the classical tetraterpenoids, that is, C40 carotenoids, whereas *apo-carotenoids* are the resulting compounds from carotenoid metabolism that shortens the C40 structure of the parent compound (carotene or xanthophyll), while the biosynthesis of C30 carotenoids has been described in bacteria. The lipophilic nature of these compounds, with the resulting attracting yellow to red colours derived from the extensive chain of conjugated double bonds, is the main feature that specifies the modes of interactions with other biomolecules and the environment where these processes take place. Thus, in fruit and vegetal tissues, carotenoids are biosynthesized in plastids which present their own organelle membrane and behave in a lipophilic environment with other biomolecules. Consequently, the subsequent tissues where carotenoids may incorporate and develop further activities should resemble in a similar way either a membrane macrostructure or a lipophilic surrounding.

Carotenoids significantly contribute to the nutritional value of natural sources where they occur including fruit and vegetables, algae, eggs and fish [1]. This is due to the exclusive dependence of animals on diet to incorporate these compounds to inner tissues and systemic circulation where they develop significant functions and biological activities. Around 10% of the described carotenoids in nature present the structural requisites to be metabolized in vitamin A [2]. In addition, all carotenoids exert important functions in immunity, participate in the antioxidant defence system and are related with a reduced risk of developing chronic diseases [3,4]. These biological activities are the elements to provide evidence of the inverse association between the intake of carotenoid-containing fruits and vegetables or elevated serum carotenoid levels with risk for various chronic diseases [5,6,7,8,9,10].

**Figure 1:**
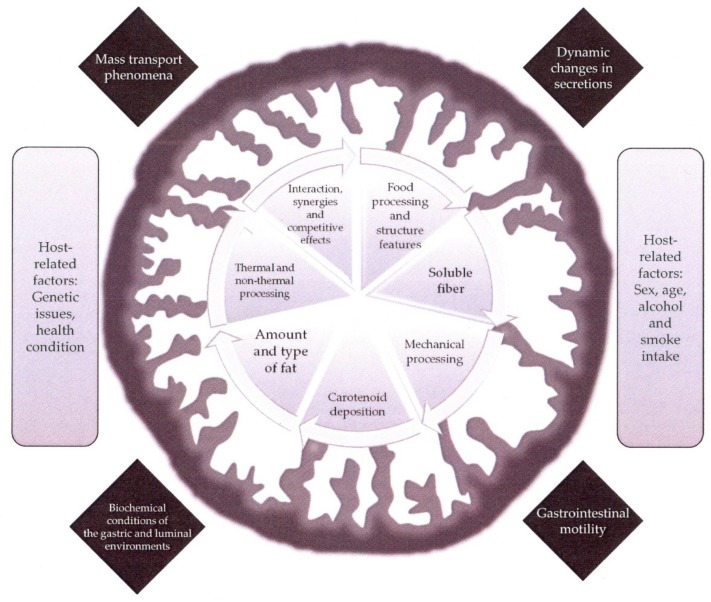
Multifactorial weights in bioaccessibility of carotenoids, including exogenous factors (denoted within the inner circle), physiological issues (indicated in the black diamonds) and host-related factors (stated in the grey columns).

In comparison with the attained knowledge regarding the structural features, physio-chemical properties and biological functions and actions of the carotenoids biosynthesized in terrestrial organisms, the progress made with the carotenoids from marine surroundings advances with a lower rate so far. This circumstance is at least surprising considering that the highest structural diversity is found in the carotenoids from marine environments, even some of them (the acetylenic carotenoids) seem to be restricted to aquatic sources [11]. The puzzling structural arrangements arise from the lively biosynthetic micro- and macro-algal species and, to a lower extent, from photosynthetic and non-photosynthetic marine bacteria. From these organisms where the de novo biosynthesis starts, two additional steps of structural transformations are possible, one in the herbivorous animals that live in symbiosis with the former microorganisms and show active transformation routes for carotenoids and subsequently in the carnivorous animals [12]. This attractive natural product chemistry has been the subject of interesting reviews [12,13,14,15], which suggest the existence in marine animals of further new structural arrangements with interesting resulting functions, as well as metabolic pathways coupled with their enzymatic systems that need to be unravel. Although those issues will provide new research topics in the upcoming future, it should be noted that some constraints apply to the idea of incorporating new carotenoid structures to our diet because of enhanced health properties or even new ones. Thus, the practical convenience of the marine source where such novel assemblies are biosynthesised, the toxicological issues both of the ‘new bioactive’ and the matrix and the bioaccessibility and bioactivity in vivo are boundaries that arise in this prospect. Therefore, within the group of ca. 40 different carotenoids that we can obtain from the diet, marine carotenoids limit to fucoxanthin and astaxanthin and their corresponding metabolites that accumulate in the edible tissues of aquatic animals, algae or in microorganisms. In addition, seafoods are also a potential source of β-carotene, β-cryptoxanthin, lutein and zeaxanthin although those foodstuffs find straight competitors in the conventional fruits and vegetables. In addition, a current key piece of knowledge to establish the actual contribution of carotenoids to human health is the study of the bioaccessibility. This concept observes the entire sequence of events that take place during the digestive transformation of food into material that can be assimilated by the body, the absorption/assimilation into the cells of the intestinal epithelium and the pre-systemic metabolism [16]. The factors affecting bioaccessibility of carotenoids are grouped within food structure, food matrix and processing features, physiological issues and genetic and host-related aspects, which have been the common thread in recent reviews [17,18] and are depicted in Figure 1.

Taking both topics into account, this review comprehends those significant contributions made to date regarding the bioaccessibility issues of those marine carotenoids we can readily obtain from available dietary sources or from supplements. In vitro experiments and in vivo studies, either with animal models of with humans applied to fucoxanthin and astaxanthin have provided several hints of their absorption, metabolism and tissue distribution. Hence, pharmacokinetic parameters and some transformation routes have been unravelling or at least tentatively denoted and a significant amount of data has been produced, which it is evaluated in this revision. References to other carotenoids that we can obtain from marine source but that have their ‘terrestrial sources’ counterparts are also acknowledged. Finally, and considering the meaning of aquaculture practice and that bioaccessibility of marine carotenoids to farmed animals is a topic that is in continuous progress, a section is focused on bioaccessibility of marine carotenoids in aquaculture.

## 2. Bioaccessibility of Fucoxanthin

**Figure 2:**
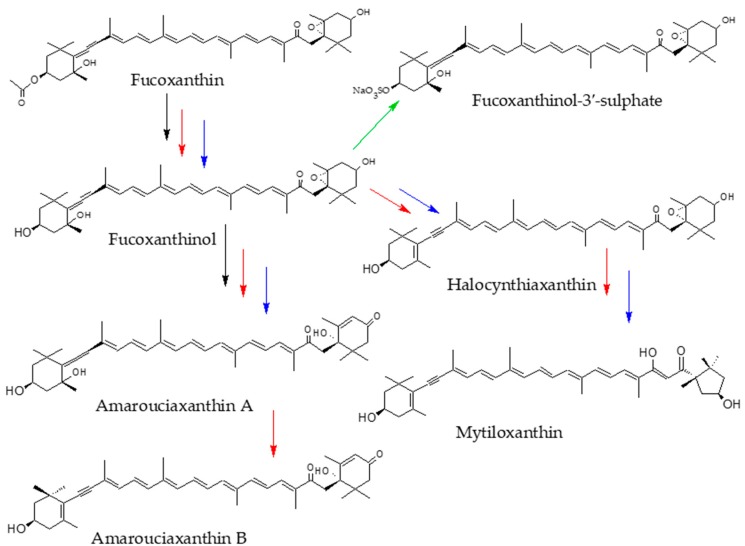
Metabolic pathway of fucoxanthin in different animal species. Arrows mean metabolism in mammals (black), bivalves (blue), tunicates (red) and hens (green).

Fucoxanthin is one of the most abundant carotenoids in nature, with an estimated contribution higher than 10% of the total carotenoid production, mostly in the marine environment [19]. This orange carotenoid is present in the Chromophyta family, including Phaeophyceae (brown seaweeds) and Bacillariophyta (diatoms) and it features a distinctive structure with an allenic carbon, a 5,6-monoepoxide function and nine conjugated double bonds (Figure 2). Fucoxanthin has attracted the interest of numerous researchers due to its considerable potential and promising applications in human health. Several of the health-promoting effects of fucoxanthin are due to the uncommon allenic carbon, which promotes the radical scavenging activity [20]. Protective effects in different human tissues have been recently reviewed for this carotenoid [21] including liver, skin, cerebrovascular, bone and ocular well-being, in addition to the demonstrated antiangiogenic, anti-inflammatory and anticancer activities [22]. Consequently, fucoxanthin is the bioactive used in many commercial nutritional supplements worldwide. But, undoubtedly, the most promising properties are the implication in the anti-obesity metabolism and the antidiabetic activity [23,24,25]. Indeed, the supplementation of fucoxanthin in adults matching with an increase in serum fucoxanthinol was correlated with a significant reduction on HbA1C serum levels (a biomarker of blood glucose levels) in subjects carrying a specific allele on the uncoupling protein 1 gene, which encodes a thermogenic protein that dissipates glucose and lipids as heat [26]. Those data are on the way to obtain a tailored application of fucoxanthin-based nutraceuticals as a strategy for the population genetically at high risk of developing diabetes. However, to successfully achieve that aim, it is necessary to consider the bioaccessibility data obtained for fucoxanthin so far, both from in vitro experiments and in vivo studies, so that the extent of those bioactive properties could be evaluated according to the bioaccessible amount.

In this sense, in vitro experiments allow the association of factors that impact in bioaccessibility, which measurement is not feasible by in vivo approach. The former consists in the application of in vitro digestion protocols coupled with the differentiated Caco-2 cellular line, one of the most widespread models for mimicking the absorption of bioactive compounds by human intestinal epithelium. Hence, the in vitro cellular absorption of 11 carotenoids solubilized at the same concentration in mixed micelles was determined with this cellular line [27]. The application of the same experimental conditions showed that although fucoxanthin was effectively accumulated by the Caco-2 cells, its absorption rate was the lowest among the rest of carotenoids and similar to that of neoxanthin. Subsequently, with that in vitro approach, it was observed that during the fucoxanthin absorption by Caco-2 cells, this carotenoid was hydrolysed to fucoxanthinol [28], so that the small intestine is the main tissue for the in vivo hydrolysis of fucoxanthin. Indeed, further studies demonstrated the transformation of fucoxanthinol into amarouciaxanthin A in human hepatoma HepG2 cells [29]. This process requires NAD^+^ as cofactor and consequently it is necessary the participation of some enzyme(s) to achieve this transformation.

Several attempts have been done to establish the complete cleavage of fucoxanthin to retinoid-like molecules during the in vivo metabolism in animals [30]. In bivalves, [15,31] three different metabolites have been identified: halocynthiaxanthin, amarouciaxanthin A and mytiloxanthin (Figure 2, blue arrows). In tunicates amarouciaxanthin B has also been identified [32]. A complete review of the fucoxanthin metabolites identified in marine organisms has been published by Maoka [12]. In rodents, only fucoxanthinol and amarouciaxanthin A (Figure 2, black arrows) have been identified in vivo, according to the in vitro characterization noted above [28,29,33]. Other catabolites could be produced, although the identification has not been completely performed [34]. Thus, the first assay of in vivo bioaccessibility of fucoxanthin was achieved in laying hens fed with a diet containing 15% of brown algae [35]. The presence of metabolites was measured in the egg yolks, with fucoxanthinol and fucoxanthinol-3′-sulphate (Figure 2, green arrows) as the single derivatives with no trace of fucoxanthin, what leads to assume that the parent compound could be de-acetylated in the intestinal lumen and then transported through the blood to inner tissues.

The pharmacokinetic parameters of fucoxanthin and its metabolites have been calculated after a single intragastric administration of 0.105 mg (160 nmol) to 6 mice [36]. The parent compound was not detected in blood or in any of the tissues. On the opposite, fucoxanthinol and amarouciaxanthin A reached a postprandial peak after 4 h and then gradually decreased for 24 h in plasma, erythrocytes, liver, lung, kidney, heart and spleen. Interestingly, the concentration of the metabolites reached the maximum value in the adipose tissue after 24 h and they were still detectable after 72 h. The maximum concentration (Cmax) for both metabolites were quantified in the liver (584 and 190 nmol/g), while the adipose tissue showed the lowest values, although the area under the curve to infinity (AUC∞) of fucoxanthinol showed the highest value in the liver while for amarouciaxanthin A the maximum value was observed in the adipose tissue. The results indicated that most of the fucoxanthinol could be metabolized to amarouciaxanthin A in the liver during the initial 24 h, whereas the same process takes place in the adipose tissue with a slower conversion rate. In fact, a study with obese/diabetic mice model showed that more than 80% of the fucoxanthin metabolites were accumulated in the white adipose tissue [37]. This specific accumulation of fucoxanthin metabolites (mainly amarouciaxanthin) in the adipose tissue has been later confirmed [34] with fucoxanthin-supplemented feed (5.18 g/d), corresponding to 0.128 mmol fucoxanthin/d for 14 d. Interestingly, those authors developed the same assay in parallel with a lutein-esters supplemented feed and the metabolites accumulated preferentially in the liver and plasma instead of the adipose tissue. Consequently, the tissue distribution of fucoxanthin metabolites is not associated with its lipophilicity. The distribution of fucoxanthin and their metabolites obtained from different studies is summarized in Table 1.

In contrast with the assumed theories that point to a low bioaccessibility of fucoxanthin, the comparison of AUC∞ of fucoxanthinol and amarouciaxanthin A with that of astaxanthin [42] shows that the ratio of absorbed fucoxanthin to the dose is higher than the value observed for astaxanthin [36]. Indeed, the comparison of levels from mice plasma after 2 h of administration of purified carotenoids confirms that fucoxanthin is absorbed in a similar fashion to β-carotene or lutein [29,43,44]. Even it has been shown that the administration of lutein esters at 20-times the amount of fucoxanthin did not imply a higher accumulation in mouse tissues [34]. Even more, those authors compared the absorption between all the metabolites in the different tissues and plasma and they concluded that fucoxanthin is readily more efficiently absorbed than lutein esters.

Another expected idea is that fucoxanthin would be absorbed without conversion to any metabolite only when the amount of daily ingested fucoxanthin surpasses the hydrolytic activity at the intestine. To test this hypothesis, Hashimoto et al. [36] developed an experiment with mice (*n* = 8) providing a daily intake of 160 nmol of micellar fucoxanthin for 7 days and took the samples for analysis 24 h after conclusion of the supplementation period. Only then, fucoxanthin was quantifiable in erythrocytes and in all the analysed tissues, except in plasma. This was the first report describing the accumulation of intact dietary fucoxanthin in mammalian tissues. Later studies with rats [40], were also able to detect fucoxanthin in plasma after the intake of 65 mg/kg body wt. with a peak concentration (29.1 µg/L) at 7.7 h.

Surprisingly, few studies have dealt with the bioaccessibility of fucoxanthin in humans (Table 2). Asai et al. [45] estimated the intestinal absorption of epoxyxanthophylls, evaluating the plasma concentrations of fucoxanthin and its metabolites before and after one-week of dietary supplementation with the brown macroalgae wakame (*Undaria pinnatifida*), which provided 6.1 mg (9.26 µmol) of fucoxanthin. The results suggest, in agreement with previous studies [46,47] that the intestinal absorption of epoxyxanthophylls is rather limited in humans. Specifically, 0.8 nmol/L of fucoxanthinol were quantified in plasma, which was below the limit of quantification, while fucoxanthin and amarouciaxanthin A were absent. On the opposite, other carotenoids such as lutein or β-carotene increased their concentrations in blood. Several hypotheses could explain the low bioaccessibility of the epoxyxanthophylls, including the presence of dietary fibres in the food matrix [48], a faster first-pass metabolism by the detoxification enzymatic systems after the intestinal uptake [45], or a lower affinity of the intestinal transporters for the less lipophilic epoxyxanthophylls.

More recently Hashimoto et al. [49] have determined the human pharmacokinetics of fucoxanthin (31 mg) in eighteen volunteers with similar results as the noted above: only fucoxanthinol was found in plasma but not fucoxanthin nor amarouciaxanthin A. The maximum concentration (44 nmol/L) was reached at 4 h and then decreased until 24 h of monitoring, yielding an AUC∞ value of 663 nmol/L×h. The authors concluded that the bioaccessibility of fucoxanthin in humans seems to be lower than that of other carotenoids (β-carotene, lutein or astaxanthin) with AUC values in the µmol/L×h range, although higher than in mice [36].

In this sense, there is still no direct evidence of the identification of any specific intestinal transporter for fucoxanthin, although considering the obvious structural similarity with other carotenoids, the SR-B1 has been proposed as a plausible protein-type receptor [48]. In this line, several indirect reports support this proposal. For example, Wu et al. [50] have reported an increase in mRNA expression levels of a transcriptional factor (PPARγ) that activates carotenoid transporters (as SR-B1) in adipose tissue when mice were fed with dietary fucoxanthin. And more recently, Ravi and Baskaran [51] correlated the increase in the bioaccessibility of fucoxanthin in a rat model with increases in the expression of PPARγ and SR-B1. In addition, the excretion levels to the intestinal lumen could explain one of the features of the fucoxanthin malabsorption in humans, by the activity of the multi-drug resistance 1 as an efflux pump [48].

Due to the low human bioaccessibility of fucoxanthin, different strategies have been developed to increase its absorption efficiency. The encapsulation of this bioactive carotenoid in liposomes, micelles or nanogels is a common practice to deliver it to the body [52,53]. Specifically, it has been shown that in vitro bioaccessibility of fucoxanthin improves from 21.5% in control samples to 68% in chitosan-glycolipid hybrid nanogels [51,54]. Even more, the encapsulation of fucoxanthin in chitosan nanoparticles coated with casein increase the AUC∞ value [55]. Other alternatives are nanoemulsions, that is, small lipid droplets (<100 nm) dispersed in water, which enhance the incorporation into mixed micelles and therefore the assimilation in the gastrointestinal tract [56,57]. More recently, Kotate-Nara and Nagao [58] have proposed that the presence of digalactosylmonoacylglycerol and sulfoquinovosylmonoacylglycerol increases not only the uptake but also the transport of fucoxanthin through the Caco-2 cells, because the lysoglycerolycolipids can decrease the tight junction integrity, increasing consequently the accessible surface area of the lateral side of the cell membrane. A novel trend with excellent results in terms of bioaccessibility efficacy is the reinforcement of dairy foods with fucoxanthin [41]. Hence, the fucoxanthin-fortified whole and skimmed milk provided a higher accumulation of the fucoxanthin metabolites (fucoxanthinol and amarouciaxanthin A) in plasma and several tissues of the mice model in comparison with the control group that was fed with microalgae powder.

## 3. Bioaccessibility of Astaxanthin

**Figure 3:**
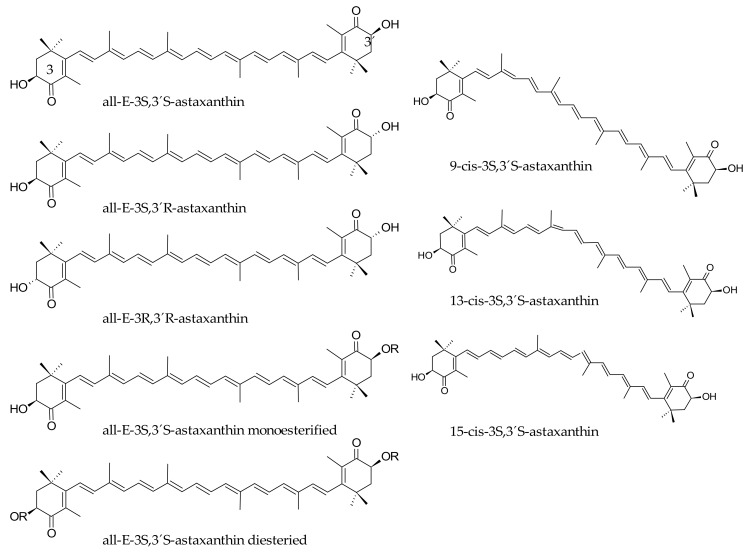
Structural arrangements of astaxanthin isomers.

The red carotenoid astaxanthin is structurally characterized by the presence of two β-rings with two pairs of hydroxyl and keto groups at the C3/C4 and C3′/C4′ positions (Figure 3). This feature produces that in addition to the possible cis-trans isomers, two additional enantiomers (3S,3S′) and (3R,3R′) and one mesomer (3S,3R′) are produced from the alternative positions of the hydroxyl groups at the C3/ C3′ carbon atoms, although the (3S,3S′) is the most abundant isomer in nature [59]. As stated before, animals depend on the diet to systemically incorporate carotenoids, which are biosynthesized in the vegetal kingdom, bacteria and fungi [60]. Among them, the green microalgae *Haematococcus pluvialis* shows the highest biosynthetic capability and accumulation of astaxanthin in nature [61], while some planktonic microcrustaceans are also a significant source of this ketocarotenoid. Thus, several animal species bioaccumulate this pigment as they consume significant amounts of those astaxanthin sources. This is the case of some birds (flamingos and quails) that accumulate this pigment in the feathers, the skin, the integument and inner tissues, whereas astaxanthin is the carotenoid responsible for the deep red or pale pink hues of the shell and meat of several aquatic animals including salmonid species, shrimps, lobsters and crayfish [62,63].

A significant amount of experimental evidences is available regarding to the potential benefits of astaxanthin in human health [60,64,65,66,67]. The application of different test models has demonstrated that astaxanthin could be considered as a ‘super-antioxidant’ with the strongest antioxidant activity in comparison with other carotenoids [68], vitamin E and vitamin C [69]. Supplementation of the diet with astaxanthin sources leads to a higher antioxidant capacity towards reactive oxygen species [70,71,72,73,74]. The consistency of these results points to assign a remarkable potential to astaxanthin as a bioactive compound that may decrease the risk of developing adverse diseases including cancer [67], gastric ulcers by *Helicobacter pylori* [75] and other gastrointestinal disorders [76,77]. Some studies claim the positive influence of astaxanthin in the gastrointestinal health [70,78] and as a therapeutic agent against cardiovascular disease [79,80,81,82]. The antioxidant activity of astaxanthin presents other collateral benefits in the enhancement of the immune system as it has been shown by in vitro and in vivo experimental animal models [65] and humans [83]. Diets rich in astaxanthin sources could be beneficial for population at higher risk of heart attacks, considering the evidences obtained on the inhibition of LDL oxidation by astaxanthin what may link the consumption of marine products with the prevention of atherosclerosis [84]. In addition, astaxanthin reduced triacylglycerides and cholesterol in VLDL [85] and increases the basal artery blood flow [86]. Other studies have explored further effects of astaxanthin like the protection against toxicity of ethanol and drugs [87], the enhancement of condition of ocular tissues [88,89,90] and skin [72,91] and improvement of fertility [60,92,93]. Another promising application of astaxanthin is related with development of skincare and cosmetic products to prevent the deleterious effects of exposure to UVB and UVC [94] and the treatment of pigmentation patches and marks [95].

Considering the potential bioactivity of astaxanthin, a significant research attention was driven to determine the bioaccessibility of this pigment. Cell culture models based on the Caco-2/TC7 cellular lines have been applied to obtain the efficiency of the absorption of astaxanthin from different sources [96] as well as in vivo experimental models with animals (rats, mice, dogs and cats) [83] and humans [83,97,98,99,100]. These trials in humans were designed as small interventions studies and used different sources of astaxanthin (wild or farmed salmons, *H. pluvialis*, or synthetic astaxanthin) and different intake routines (single or repeated doses).

The bioaccessibility of astaxanthin from fish was determined in a randomized double-blind trial in 28 volunteers [97]. Participants ingested 250 g of wild or farmed salmon containing 5 µg of astaxanthin per gram. The source of astaxanthin in the wild salmons is krill whereas feed supplemented with synthetic astaxanthin is the pigment source for salmon from aquaculture. At 3, 6, 10 and 14 d, the plasma levels of astaxanthin were higher in those volunteers ingesting aquaculture salmon in comparison with the wild-salmon diet group. Interestingly, the ratio of the (3S,3′S) isomer to the rest of astaxanthin isomers was higher in plasma than in the salmon flesh. This fact suggests that isomerization is an influencing factor of the bioaccessibility efficiency of astaxanthin. According to this result, Coral-Hinostroza et al. [99] showed the effects of administering a purified chiral isomeric mixture of 10 mg and 100 mg astaxanthin diesters as dressing for a pasta salad. Astaxanthin plasma elimination half-life was 52 (SD 40) h and there was a non-linear dose response and selective absorption of cis-isomers.

The study published by Osterlie et al. [98] gave significant insights of the pharmacokinetics of astaxanthin. They reported a change in the plasma concentration of astaxanthin following the administration of a single 100 mg dose of a racemic astaxanthin mixture in olive oil-pearls. The authors examined the appearance and distribution of astaxanthin isomers in the lipoprotein fraction of plasma from three males. The concentration of astaxanthin reached a peak at 7 h (1.3 mg/mL) while the pigment was observed for 72 h. Additionally, 47% of the astaxanthin was selectively accumulated the VLDL-containing chylomicra, whereas 29% and 24% distributed within LDL and HDL, respectively. Therefore, the study showed that oral administration of a mixture of *cis-trans* astaxanthin isomers leads to a significant raise of plasma levels, without any appreciable metabolic transformation in contrast with previous reports.

Odeberg et al. [42] determined the pharmacokinetics of astaxanthin from a different marine source. A single dose of the green microalgae *Haematococcus pluvialis* dispersed in a lipid-based formulation or as a commercially available food supplement, both containing 40 mg of astaxanthin was administered to a group of volunteers. The bioaccessibility of astaxanthin was enhanced in those individuals who received the lipid-based formulation, ranging from 1.7 to 3.7-times that of the commercial formulation. The lipid-based formulation was produced with a high content of the synthetic hydrophilic surfactant polysorbate 80 (B) while the commercial formulation was based in a dextrin gelatin. The accumulation of astaxanthin in the plasma of the volunteers reached a maximum amount of 0.192 mg/L, a figure which it is significantly lower than the result by Osterlie et al. [98] cited above. It is not possible to underline a single reason for such different result. Astaxanthin was given either free or esterified in those studies and the chemical properties of the lipid-based formulation were also dissimilar. Nevertheless, both studies allow to note that quality control of the supplements available in the market must be performed, as the consumers should know not only the bioactive facts of the supplement but also its nutritional efficacy in terms of bioaccessibility [16]. Indeed, the bioactivity resulting from the extent of bioaccessibility would be unlike. Hence, recent studies have shown that the antioxidant properties of astaxanthin were improved in rat plasma and liver tissues after administration of biomass from *Haematococcus pluvialis* dispersed in olive oil [101,102,103].

At this point it is necessary to note again that bioaccessibility of carotenoids is substantially affected by a multifactorial group of effects, that reach host-related conditions including sex, age, obesity, smoking, alcohol consumption (Figure 1). In this sense, Okada et al. [100] investigated the pharmacokinetics of astaxanthin after the administration of an extract from *Haematococcus pluvialis* to smokers and non-smokers, either before or after meal intake. The amount of astaxanthin supplemented was 48 mg, provided as free form. The pharmacokinetics of astaxanthin in fasted conditions was significantly faster in comparison with the one obtained in the fed state. The peak concentration was reached at different times after supplementation (8 h or 24 h in fasted and fed conditions, respectively), so that the values of the postprandial area under the curve and, consequently, the efficiency of the bioaccessibility was higher in the fed state. The reason for the increase in absorption of astaxanthin could be the presence of fat that stimulates the excretion of bile. Enough amounts of bile help the dispersion of carotenoids in the digestive tract, which then leads to an effective absorption of carotenoids [104]. The mean astaxanthin content in the fed state group (0.11 mg/L) is in the same range to the result obtained by Coral-Hinostroza et al. [99] (0.28 mg/L) and correlates with the different amount of astaxanthin ingested by the volunteers (48 mg vs. 100 mg). Regarding the smoker/non-smoker effect, the authors pointed to a faster metabolism of astaxanthin in the group of smokers, that also showed a higher interindividual variability of the data. The study of Galan et al. [105] showed that smokers usually contain lower carotenoid levels in plasma than non-smokers. It has been shown that an abundance of free radicals in cigarette smoke could induce oxidative stress in both the respiratory and the circulatory system [106], so that the faster astaxanthin metabolism in the smokers should be a consequence of the astaxanthin interaction with reactive oxygen species.

**Figure 4:**
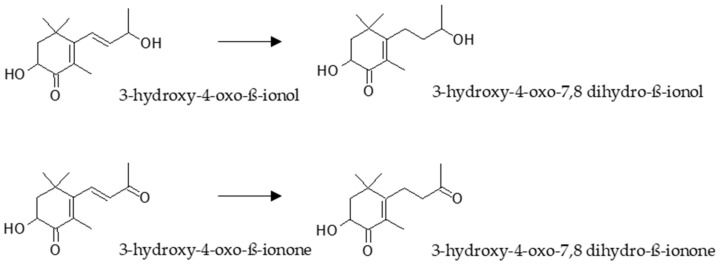
Metabolites identified during the metabolism of astaxanthin in rats and humans.

Despite all the research developed so far with astaxanthin, only four metabolites have been characterized as a consequence of its metabolism (Figure 4). In primary cultures of rat hepatocytes obtained from animals pre-treated with astaxanthin, two metabolites were characterized by GC-MS [107]. The structures were confirmed by the analyses of synthesized standards as 3-hydroxy-4-oxo-β-ionone and its reduced form 3-hydroxy-4-oxo-7,8 dihydro-β-ionone. Later, these metabolites were identified in human plasma from two volunteers who had orally taken 100 mg of astaxanthin during 24 h [108]. In addition, in the same study, other two metabolites were also identified by CG-MS of radiolabelled compounds as 3-hydroxy-4-oxo-β-ionol and its reduced form 3-hydroxy-4-oxo-7,8-dihydro-β-ionol. Interestingly, the authors showed that cytochrome P-450 system was not implicated in the metabolism of astaxanthin, neither in rats nor in human hepatocytes.

In summary, after all the studies carried out regarding to the plasma values and the distribution of isomers of astaxanthin in humans [83,97,98,99,100], some common conclusions could be noted. First, a non-linear dose-response for plasma concentrations of astaxanthin in human subjects is observed, probably due to a response mechanism to the supra-physiological dose. In this scenario, the bioaccessibility is mainly affected by the esterified/free form of astaxanthin, whereas the selective accumulation of the *cis* isomers is taking place. Among both factors, there is a scientific controversy on the bioaccessibility of astaxanthin esters. Esterification of xanthophylls is a common process in nature [109,110] and most (90%) of the natural astaxanthin in marine organisms is esterified [103]. The hydrolysis of xanthophyll esters in the gut has been denoted as a prerequisite for their absorption by the intestinal epithelium, because the ingestion of food sources rich in xanthophyll esters led to appearance of only the corresponding free forms in plasma [101,111,112], while trace amounts of xanthophyll esters appeared in that tissue [113,114]. Specifically, both the supplementation with astaxanthin esters from extracts of *Haematoccocus pluvialis* [42,109] or with synthetic esterified astaxanthin [99] have confirmed this issue of the studies noted above. Existing evidence points to an enzymatic cocktail as the responsible for the hydrolysis of xanthophyll esters in the gut, including a carboxyl hydrolase ester secreted by the pancreas [115], enzyme(s) located in the membrane of the enterocytes and those acting during packing of chylomicra, a process that may compete with the re-esterification activity that has been observed by Sugawara et al. [112]. Recently, it was shown through an in vitro study with the Caco-2/TC7 cellular model that micellarized esters of astaxanthin were partially absorbed, while the free form was absorbed almost completely [96].

Finally, there is a global trend towards the inclusion of natural ingredients in all forms of food products, as well as in the nutraceutical and cosmetic formulations, what arises from the increasing concerns for consumer safety and the regulatory issues about the presence of synthetic chemicals. In the case of astaxanthin, the demand for a natural sourced has launched the *Haematococcus pluvialis* algae in the global market. Hence, the number available products based in the potential bioactivity of astaxanthin with many applications is increasing, with most of the products marketed in the form of soft capsules. Currently there is a revision by some international regulatory bodies of the ADI values for astaxanthin, which were set at 2.4 mg/d [116]. Although it seems that the efficiency of bioaccessibility is not affected by the natural or synthetic origin, a different scenario is established when bioactivity data are considered. The results of a recent study show that dose level of natural astaxanthin to reach the same antioxidant activity than the synthetic alternative is 20–30 times greater [117].

## 4. Bioaccessibility of Other Marine Carotenoids

The range of applications of microalgae as source of carotenoids is not limited to astaxanthin-based products but also to the manufacture of supplements with biologically active carotenoids like β-carotene, lutein and zeaxanthin with significant contributions to human health [118] that enter in the nutrition and supplement market, which is expected to reach 220 $ billion globally in 2022. Although consolidated regulations regarding the safety, labelling and market issues exist in most countries, aimed to keep public health questions and guarantee the quality of this kind of products, there is a lack of research in the nutritional efficiency of the micronutrients and bioactive compounds contained in the supplements. Consumers depend on labelling to know the amount of the component(s) intended to supplement the diet but that amount may significantly differ from the quantity that is absorbed and reaches the systemic circulation, that is, bioaccessibility is not measured while it is assumed that there is equivalence between the bioactive content and the biological relevance [118]. However, the veracity of that equivalence is unclear and the recent scrutiny of bioaccessibility through in vitro protocols has started to show that there is a full catalogue of factors that drive the efficiency of the digestion and absorption processes. These factors include host-related physiology and metabolism that it is highly variable between subjects, characteristics of the co-ingested food with the supplement that promote interactions either positive or negative in the final digestion/absorption outcome and factors related with the physico-chemical properties of the bioactive as well as the featured matrix where it is formulated. Hence, it has been shown that the human plasma response for β-carotene after the three-days ingestion of *Dunaliella salina* gel caps was significantly lower than the observed when water-dispersible synthetic β-carotene beadlets were ingested for the same period [119]. The carotenoids of the microalgae *Scenedesmus almeriensis* showed a poor in vitro digestibility (<1% of the total content were incorporated into mixed micelles), so that the direct consumption of the biomass is not the suitable way to obtain a significant absorption [120]. Following, the authors described that once the biomass was extracted with olive oil, almost 90% of the carotenoid content was successfully transferred to the micelles in vitro, pointing to the exceptional influence of the lipid environment in the efficiency of bioaccessibility of carotenoids. Such strategy was applied to measure the in vivo bioaccessibility of β-carotene, astaxanthin and lutein from algae biomass of *Spirulina platensis*, *Haematococcus pluvialis* and *Botryococcus braunii*, respectively in rat model. The postprandial plasma responses were significantly different for astaxanthin that accumulated higher than β-carotene and lutein, although these carotenoids also showed enough circulating levels in plasma to consider the corresponding olive oil extracted biomass as a suitable source [101].

## 5. Bioaccessibility of Carotenoids for Aquaculture

Nutrition is among the foremost contributors to achieve successful physiological and morphological changes of aquatic species during rearing that finally drive to an economically effective aquaculture practice, providing the consumers a first-rate product in terms of organoleptic properties and texture quality. Therefore, many of the advancements made in aquaculture are related with nutrition and feed processing, as well as in the organization and operability of feed facilities. Rearing fish and crustaceans in captivity, which owe their colour to carotenoids means that diet must be supplemented with these pigments to reach and even improve the colouring levels of the animal tissues [121]. Application of carotenoids in aquaculture covers intensive production of shrimp and prawn, salmonids and some ornamental fish species, what represents a small fraction of the total aquaculture industry [122]. However, while the fish meal represents ca. 60% of the total feed cost [123], carotenoids are one of the feed ingredients with the highest impact in the latter (15–20%) and they represent 6–8% of total production cost [124]. Particularly in salmonid species, the low bioavailability of carotenoids, 85–90% of the ingested carotenoids do not deposit in the flesh [125,126,127] and the consumer preference for salmonids with deep pink colour, because this is associated with superior flesh quality [128], are the causes to place the “colouring matter” in the top cost-effective questions to be approached in aquaculture. Indeed, carotenoids are more than just colour because several biological effects have been observed to claim their role in salmonids and other aquatic species as immune-enhancers, influencing positively growth performance and health condition, fertilization and egg survival and other activities like antioxidant action and protective agents towards UV-light [123,129,130,131,132], although not all the evidences allow to draw a conclusion.

Within this scenario, specific efforts have been made to investigate the factors influencing bioavailability of carotenoids in aquatic species following a similar strategy as that applied to establish the carotenoid bioavailability in mammals, resulting in a list of effectors that contribute to the carotenoid absorption and the final deposition of the feed pigment(s) in the tissues. The list includes factors like the carotenoid type and origin (either natural or synthetic astaxanthin/canthaxanthin), dietary source (natural feed or formula) and dose levels [122,133,134]. Also, genetics has been reported to change the metabolism and ability of disposition of carotenoids in aquatic species, while the amount and origin of fat and co-supplementation of the feed with other lipophilic antioxidants have revealed to affect carotenoid bioavailability to some limited extent [129,135]. In addition to these exogenous factors related with food matrix and structure, physiological issues might play a significant role in the limited bioavailability of carotenoids in aquatic animals. Hence, although efficiency of carotenoid bioaccessibility from the gut is relatively low, with an extensive amount of the feed carotenoids remaining in the gastrointestinal tract, the grade of enzymatic hydrolysis reached when carotenoids are supplied in the ester form [136,137], the carotenoid metabolism in liver and subsequent excretion to the gut [138,139], the conversion to uncoloured metabolites [140,141,142] as well as the low transport rate values are physiology-related factors difficult to change, what hindrance even more the bioavailability of carotenoids in aquatic animals. Indeed, it has been suggested that other routes different to oral administration may bypass the substantial number of factors and their significant limiting effects in carotenoid bioavailability [143]. Finally, the recent discoveries that have changed our picture of the mechanisms for the intestinal absorption of carotenoids, with the implication of protein-type transporters that facilitate diffusion of carotenoids through the apical side of enterocytes might create new research lines aimed to include genetic traits controlling (upregulation) the synthesis of those protein-type transporters.

Synthetic astaxanthin, canthaxanthin or another suitable precursor are the starting alternatives in aquaculture to provide colour to the aquatic species. The pigments are supplied as water-dispersible gelatine beadlets [144,145], which are mixed with the rest of the basal feed and then pelleted. Commonly, the pigment concentration in the feed is within the 37–75 mg/kg-dry-diet range [146] to achieve pigment content in the tissues around 6–12 mg/kg. The considerable expense [147], the regulations concerning use of synthetic products in the food chain, as well as the consumer preference of natural products over those from chemical synthesis, promoted the search for alternative natural colouring sources. Thus, oil extracts from marine sources like krill, crayfish or red crab and extracts from by-products originated at the processing industry of these crustaceans; extracts from biomass of the Chlorophytes *Haematococcus pluvialis*, which can accumulate 30 g of astaxanthin per kg of dry biomass, or *Dunaliella salina*, the reference source for β-carotene and extracts from biomass of yeasts, [66] started to enter in the feed-fish market to compete with the synthetic carotenoids (Table 3).

Production of synthetic astaxanthin is estimated to be about 880–1000 $/kg [156] and the market price could reach 2000 $/kg [64]. These values are higher than the production and market costs of natural astaxanthin from biological sources. Thus, production cost of astaxanthin from *Haematococcus pluvialis* has been estimated in 718 $/kg [157], although the figure is higher (1536 $/kg) in other projected scenarios due to the different cost of energy and labour [158]. Indeed, two additional factors increase the cost of natural astaxanthin in practice. Intact algae cells provide a low bioaccessibility of astaxanthin, so that the biomass should be processed before feeding to disrupt the cell walls [136,159,160]. Moreover, astaxanthin accumulates in its homo- and hetero-ester kinds and the enzymatic hydrolysis of astaxanthin esters seems to be inefficient in aquatic animals, although contradictory results have been reported [153,161,162]. Consequently, the required amount of astaxanthin from natural sources in the feed to reach the target colour in the fish would be higher in comparison with the synthetic astaxanthin.

Regarding β-carotene, this pigment stays at the highest carotenoid market share, which it is dominated by private companies that yield 90–98% of the total production from the synthetic way [163,164]. However, alternative technologies have been studied to produce natural β-carotene, some of them are still promising or achieved at a not competitive price, while biotechnological processes using microalgae have been successfully implemented [165], to go chasing a share of the natural carotenoid market that presents prices in a random range (300 $–3000 $/kg). As mentioned before, *Dunaliella salina* is the main character for production of β-carotene, considering that this pigment (a mixture of *cis* and *trans* isomers) accounts 80% of the total carotenoid content [166]. It is approved by FDA as food colour and is recognized as safe natural colour [59,167].

There is a growing diversification of the microalgae contents (particularly of the bioactive lipids) to obtain high-value products that supply the global demands of more profitable markets like the pharma-care and dietetics niches. This may undercut the progress in research of microalgal biotechnology to satisfy the production for aquaculture feeds, which is estimated in the range of tons [168] that necessarily must face several challenges that include the application of highly-rate standards in quality and safety issues and production costs [169].

## Figures and Tables

**Table 1:** In vivo bioaccessibility of fucoxanthin in rodents: Maxima concentration determined in different tissues and plasma.

Dose	Plasma			Liver			Adipose Tissue			Ref.
	F ^7^	FxOH	AxA	F	FxOH	AxA	F	FxOH	AxA	
1.5 nmol FxOH s.d. ^1^ i.v. ^2^		29.1 pmol/mL	17.2 pmol/mL		108.9 pmol/g	18.2 pmol/g				[29]
160 nmol F s.d. i.g. ^3^		132 nmol/L	230 nmol/L		584 nmol/g	190 nmol/g		39 nmol/g	84 nmol/g	[36]
160 nmol/d F 7 d i.g.		45 nmol/L	82 nmol/L	15 nmol/g	83 nmol/g	40 nmol/g	23.1 nmol/g	60 nmol/g	97 nmol/g	[36]
40 nmol F s.d. i.s. ^4^		0.4 nmol/L								[28]
0.05% F 5 w diet ^5^					78.1 µg/g protein	64.7 µg/g protein				[38]
0.128 mmol/d F 14 d diet ^5^		0.34 µmol/L	0.95 µmol/L		0.85 µmol/kg	0.96 µmol/kg		2.14 µmol/kg	7.85 µmol/kg	[34]
3.1 µmol F s.d. d.i. ^6^		0.33 µmol ^8^								[39]
3.2 µmol FxOH s.d. d.i.		0.44 µmol ^8^								[39]
2 mg/kg F s.d. i.v.	14,000 µg/L	598.2 µg/L								[40]
65 mg/kg F s.d. i.g.	29.1 µg/L	263.3 µg/L								[40]
7 mg/kg F s.d.	18.8 nmol/L	68.6 nmol/L								[41]

^1^ s.d.: single dose; ^2^ i.v.: intravenous; ^3^ i.g.: intragastrically; ^4^ i.s.: intubation in the stomach; ^5^ ”ad libitum” diet; ^6^ d.i.: duodenal infusion; ^7^ F: fucoxanthin, FxOH: fucoxanthinol, AxA: Amarouciaxanthin A.; ^8^ Detected in lymph but not in plasma.

**Table 2:** In vivo bioavailability of fucoxanthin in humans.

Doses of F ^1^ Administered	FxOH in Plasma	Reference
6.1 mg 1 week	0.8 nmol/l after 1 week	[45]
31 mg one dose	44 nmol/L at 4 hours	[49]
2 mg/d 8 weeks	2.7 nmol/L after 8 weeks	[26]

^1^ As in Table 1.

**Table 3:** Common sources of marine carotenoids applied for pigmentation of salmonid species.

Source	Carotenoid Composition	Pigmentation Species	Reference
*Marine and freshwater sources*			
krill	astaxanthin diester (200 mg/100 g oil)	Coho salmon (*Oncorhynchus kisutch*)	[148]
shrimp wastes	astaxanthin (3–12 mg/kg)	Rainbow trout (*Salmo gairdneri*)	[135]
crayfish oil extracts	astaxanthin	Rainbow trout (*Salmo gairdneri*)	[149]
red crab wastes and oil extracts	astaxanthin diester (155 mg/100 g oil)	Coho salmon (*Oncorhynchus kisutch*)	[150]
*Dunaliella salina*	β-carotene (200–300 mg/kg)	Black tiger shrimp (*Penaeus monodon*)	[151]
*Dunaliella salina*	β-carotene (50–200 mg/100 g)	Kuruma prawn (*Penaeus japonicus*, Bate)	[152]
*Haematococcus pluvialis*	astaxanthin (90 mg/kg)	Rainbow trout (*Oncorhynchus mykiss*, Walbaum)	[153]
*Haematococcus pluvialis*	astaxanthin (30 mg/kg)	Rainbow trout (*Oncorhynchus mykiss*, Walbaum)	[137]
Yeast			
*Phaffia rhodozima*	astaxanthin (55–80 mg/kg)	Rainbow trout (*Salmo gairdneri*)	[154]
*Phaffia rhodozima*	astaxanthin (40 mg/kg)	Atlantic salmon (*Salmo salar*)	[155]
